# Surgery of the Vermiform Appendix

**Published:** 1905-09-16

**Authors:** 


### SURGERY OF THE VERMIFORM APPENDIX.

**The Treatment of the Stump in Appendicectomy** has been the subject of much difference of opinion and many methods of dealing with the stump have been described. Yet Seelig[1] claims that a careful search of the literature fails to reveal a single article in which the special treatment of the stump is based on definite anatomical, pathological, or experimental data. In general the methods of treating the stump may be grouped under three heads, as follows:—1. Ligation of the base of the appendix, ablation of the organ distally to the ligature, and inversion of the stump by means of Lembert sutures passed through the peritoneum of the cæcum. In such cases the appendix stump is inverted into the wall of the cæcum. 2. Ablation of the organ without previous ligature of its base, and inversion of the stump into the lumen of the cæcum; Lembert sutures over the site of the inversion. 3. Simple ligation, ablation of the organ distally to the ligature, and cauterisation (with pure carbolic acid or actual cautery) of the stump. Seelig endeavours to show that the first and second methods are dangerous, and that the third method is not only free from these dangers, but also possesses distinct advantages. In the first method the appendix stump is invaginated into the cæcal wall. It is impossible to invaginate it into the cavity of the cæcum, because the base of the appendix has been ligated preliminary to ablation of the organ. Even if the stump has been cauterised, and thus disinfected, before inversion into the cæcal wall, the method is nevertheless a dangerous and unreliable one; for the inevitable exudate that forms is bottled up in a cavity under conditions which are particularly favourable to abscess formation. Covering the ligated stump with a previously prepared cuff of peritoneum is only a modified way of locking it up in a closed space, and is open to the same objections. In the second method the appendix stump is inverted directly into the cæcum. There is no danger, therefore, of imprisoning a stump exudate, for drainage into the cæcal pouch is complete. In this method, however, it is impossible to pass a ligature around the base of the appendix, for such a ligature would prevent complete inversion of the stump, and the result, therefore, is that the field of operation is exposed to infection by its communication with the infected cavity of the fæces-laden cæcum. There is also a danger of secondary hæmorrhage by this method, for the appendicular artery, which runs in the walls of the appendix in a fair proportion of cases, remains unligatured. The third method of simple ligature of the appendix, ablation distally to the ligature and cauterisation of the stump, is a safe and rational method. The objection that this method leaves uncovered, in the free peritoneal cavity, an infected stump is not valid, for it has been shown that when the stump is cauterised either by pure carbolic acid or the actual cautery it is sterile. The argument that an uncovered stump leads to the formation of adhesions which so frequently distress the patient after operation has also been shown to be erroneous. Simple ligation and cauterisation may therefore be said to be absolutely safe. Under certain circumstances the appendix cannot be dealt with in this simple way, and when difficulty is experienced by reason of extensive adhesions of the appendix due to the results of repeated previous attacks of local inflammation, Isaacs[2] recommends that the method of "decapsulation" as the best means of overcoming the difficulties encountered. Having located some portion of the appendix, preferably the proximal, the capsule of the appendix is incised longitudinally opposite the mesenteric attachment if possible, so as to avoid the larger blood-vessels. The white, glistening surface of the middle layer is the guide to the depth of the incisions. The incised serous coat peels off very easily, and with the handle of the scalpel or with a periosteum elevator, the core can be easily separated and raised from its bed without fear of hæmorrhage. This white core now serves as a guide to the capsule, inside of which it can be loosened for a short distance, then the capsule split for that distance, and the procedure repeated till the whole length is delivered. When the appendix is perforated the inner layers are gangrenous and the core is, therefore, likely to tear within its capsule on the least traction. In such a case, while it makes conditions somewhat more difficult, it is comparatively easy to follow up the capsule, find the other end of the torn core and proceed with the decapsulation. There will frequently be found a point of maximum adhesions, centralised usually at the point of pressure-necrosis from a fæcal concretion, or due to some other cause, and when this is reached and freed by decapsulation, the remainder of the appendix, be it either the proximal or the distal portion, may be found comparatively free and can be dealt with in the usual manner, taking it off together with its capsule after ligation of the mesenteric attachment which, at this stage, can be easily recognised. In the closure of the abdominal wall after appendicectomy, Bell[3] recommends using as sutures thin strands of the external oblique tendon, which can be easily split off. This certainly gets rid of the vexed question of infection from catgut.

[1] Amer. Jour. Med. Sci., Nov. 1904. [2] Med. Rec., April 15, 1905. [3] Brit. Med. Jour., Oct. 29, 1904, p. 1145.

